# Bone and bone marrow involvement in neuroblastoma: A case report

**DOI:** 10.1097/MD.0000000000022505

**Published:** 2020-10-02

**Authors:** Can Huang, Shayi Jiang, Xuelian Liao, Yanhua Li, Shanshan Li, Jingwei Yang

**Affiliations:** Department of Hematology and Oncology, Shanghai Children's Hospital, Shanghai Jiao Tong University, Shanghai, China.

**Keywords:** neuroblastoma, bone, bone marrow, recurrence, pediatric

## Abstract

**Rationale:**

Neuroblastoma (NB) can occur in any part of the sympathetic nervous system, and it is highly heterogeneous. Tumors that only involve bone marrow and bone lesions without solid masses have rarely been reported.

**Patient concerns:**

A 2-year-old girl child presented with recurrent fever, accompanied by pain in both lower limbs for more than 1 month.

**Diagnose:**

Bone marrow examination revealed NB cell invasion. Femoral and multiple vertebral lesions were observed by MRI, while head MRI, chest CT, abdominal CT, and pelvic CT showed no solid mass.

**Interventions:**

The child received the standard therapy for high-risk NB.

**Outcomes:**

She was sensitive to the initial chemotherapy protocol. Two years later, a bone marrow examination confirmed NB recurrence.

**Lessons:**

The prognosis of this special type of NB was not improved mainly based on common chemotherapy and local radiotherapy, and new treatment strategies should be explored.

## 1 Introduction

Neuroblastoma (NB) is the most common extracranial solid tumor in children,^[1]^ accounting for 8% to 10% of pediatric malignancies and 15% of childhood tumor-related deaths.^[2]^ Approximately 65% of its primary tumors are located in the retroperitoneum and partly in the mediastinum and neck. NB presenting with no solid masses and only showing extensive metastatic lesions is very rare. This case report is of a patient who was diagnosed with NB via bone marrow examination, and her general condition was evaluated. Extensive bone metastasis was observed, and no solid mass lesions were found in the comprehensive imaging examination.

## 2 Case report

**Figure 1:**
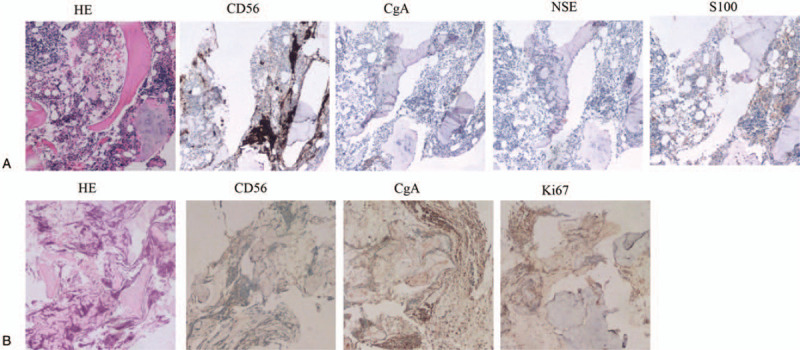
(A) Small round tumor cells expressing CD56, CgA, NSE, and S100 in bone marrow by immunohistochemistry staining; (B) small round tumor cells expressing CD56, CgA, and Ki67 in bone by immunohistochemistry staining.

A 2-year-old child was admitted to the hospital due to current fever with pain in both lower limbs for more than 1 month in March 2017. There was no special medication history and no other local or systemic symptoms, and the physical examination revealed pain in both lower limbs while walking but not limited to walking. Routine blood examination showed anemia (86 g/L), normal white blood cell counts, high erythrocyte sedimentation rate (120 mm/h), high C-reactive protein (17 ng/L), normal ferritin (247 ng/mL), high neuron-specific enolase (NSE, 70.01 ng/mL), and normal 24 h urine vanillylmandelic acid (7.7 mg/24 h). Bone marrow cytology examination showed that tumor cells accounted for 75.5% of total cells and that these cells were chrysanthemum-like. Flow cytometry showed positive expression of CD56/GD2/CD81. Histopathology of bone marrow showed positive expression of CD56, CgA, NSE, and S100 (Fig. 1A), which are specific markers of NB cells. *MYCN* examined by fluorescence in situ hybridization (FISH) was amplified in 2.6% of tumor cells. All these results confirmed NB cell invasion in the bone marrow.

**Figure 2:**
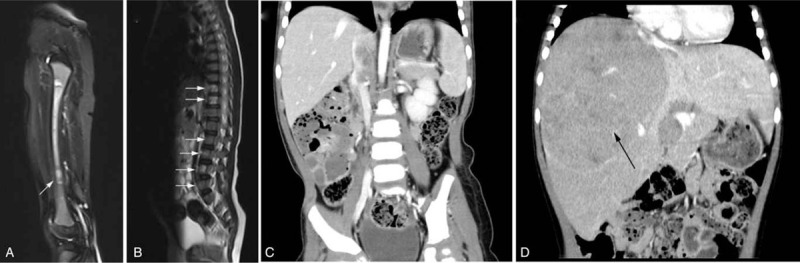
(A) Extensive hyperintensity and isointensity in the left femur by weighted MRI; (B) hyperintense in T10-11 thoracic, L2-L4 lumbar and S1 sacral vertebrae (white arrow); (C) no obvious mass was shown at diagnosis by contrast-enhanced axial CT; (D) a large heterogeneously enhancing soft-tissue mass (black arrow) in liver at relapse by contrast-enhanced axial CT.

The left thigh MRI showed a wide abnormal signal of the left femur (Fig. 2A). Thoracic and lumbar spine MRI showed that the T10-11 vertebral body density increased slightly and that the lumbosacral vertebrae had multiple instances of bone density unevenness (Fig. 2B). There was no solid tumor lesion found in the head MRI, chest CT, or pelvic abdominal CT (Fig. 2C).

To further confirm the diagnosis, a femoral biopsy of the left lower extremity was performed under general anesthesia. The intramedullary tissue structure was unclear, and the density was uneven. Postoperative pathology showed that tumor cells were seen between the trabecular bone with the tissue severely squeezed, and the cells deformed. The results suggested small-cell malignant tumors. Due to severe tissue extrusion and unclear structure, histopathological examination of the bone only showed positive expression of CD56, CgA, and Ki67 (Fig. 1B). According to the pathological results from bone marrow and bone, NB was diagnosed.

Children with no clear primary lesions or extensive bone and bone marrow lesions are not suitable for International Neuroblastoma Staging System (INSS) staging. Based on two lesions, the tumor was considered to be stage IV. In patients over 18 months of age, the patient was included in the high-risk group and received standard treatment.^[3]^ In brief, the patient received 11 cycles of induction chemotherapy, followed by tandem high-dose chemotherapy and autologous stem cell transplantation (HDCT/auto-SCT), local radiotherapy and differentiation therapy with 13-cis-retinoic acid. After 4 cycles of treatment, the bone marrow was negatively measured. After 6 months of differentiation therapy, no abnormal fluorodeoxyglucose (FDG) uptake with 18F-FDG PET-CT was observed at the final assessment. Regular evaluation was performed every 3 months.

The child complained of intermittent abdominal pain in July 2019. Physical examinations showed slight bulging of the abdomen, abdominal varicose vein exposure, and liver enlargement. The NSE level was 306.20 ng/mL. Abdominal CT suggested possible tumor metastasis to the liver (Fig. 2D). A bone marrow examination showed that the neuroblasts were tumor-cell-like (40.5%) with CD56/GD2/CD81 positivity, and histopathological examination of bone marrow showed NB cell invasion, which confirmed NB recurrence. *MYCN* examined in bone marrow cells by FISH was detected with negative amplification. Family members declined further intensive treatment and opted only for palliative therapy.

## 3 Discussion

Neuroblastoma histologically presents as a small round malignant tumor whose tumor cells originate from precursor cells of the sympathetic ridge.^[4]^ These pluripotent precursor cells can form ganglion and adrenal medulla chromaffin after migration, and these sites are also common sites of neuroblasts. Although NB usually grows in the retroperitoneal sympathetic chain or the adrenal medulla, it can be found in any part of the sympathetic nervous system and is very heterogeneous in nature. The clinical characteristics of some tumors include spontaneous remission or mature differentiation.^[5]^

It has been reported that 1% of patients have no detectable solid mass tumor.^[6]^ Reviewing the literature reports in recent years, only one patient was reported. But the clinical features, follow-up treatment and prognosis were not reported.^[7]^ In our case, the bone and bone marrow were the common sites of metastasis for NB. She had no previous medical history data and no evidence of a tumor mass present in any other part of the body. It was not clear whether natural regression characteristics of the tumor existed. The mechanism of natural regression is still unclear, but the following are considered to be associated with the spontaneous maturation of tumors: age <18 months, differentiation type, good INSS stage, non-*MYCN* amplification, TrkA expression, and apoptosis.^[8]^ Therefore, it was theoretically speculated that the natural regression characteristics of the tumor were differentiated to some extent and resulted in good outcomes. Therefore, we considered that natural regression may not explain the other primary tumor sites of the patient.

Until now, there has been no clear reference guide for the treatment of these types of NB. Based on the two sites involved and the lack of detectable primary tumor, the patients mainly relied on chemotherapy according to the guidelines for stage IV NB treatment. She experienced clinical remission at the beginning of the disease but had a poor prognosis. Primary and/or secondary resistance may occur. New treatment strategies should be explored.

Norepinephrine transporter (NET) receptors are specialized in both malignant NB and differentiated tumors. MIBG is a norepinephrine analogue that can be actively taken up by NET receptors. MIBG labelled with ^123^I has been studied in clinical trials for refractory relapsed NB.^[9]^ It is known that ^123^I-MIBG has the potential of being a powerful tool in the multifaceted approach of treatment of high-risk NB.^[10]^ Because of the intensive metastasis, the effect of local radiotherapy was limited in our patient. ^123^I-MIBG may be effective.

Many studies have shown that *MYCN* is an independent factor for unfavorable prognosis.[^11^,^12^] The key action of *MYCN* in inducing NB has been confirmed in vivo and in vitro.[^13^,^14^] The number of *MYCN* copies required to be defined as amplification is still controversial.^[15]^ For our patient, 2.6% of *MYCN* copies demonstrated triploid amplification at diagnosis, and no copies were examined upon recurrence. It is difficult to judge the status of *MYCN*. Unfortunately, other related DNA-based biomarkers were not acquired. Inhibitors of anaplastic lymphoma kinase, phosphatidylinositol-3′-kinase/Akt/mammalian target of rapamycin and other molecules implicated in the pathogenesis of NB are in I/II clinical trials.[^16^,^17^,^18^] For this type of patient, it is important to examine the expression of these molecules to improve the outcome.

High-risk NB remains a major challenge in the treatment of childhood cancer. Additionally, for NB without a detectable solid mass, current intensive chemotherapy does not improve the outcome. A deeper understanding of this special type of NB and other treatment strategies are needed to improve prognosis.

## Author contributions

**Conceptualization:** all the authors.

**Writing – original draft:** Can Huang.
